# Multiple Acrometastases in a Patient with Renal Pelvic Urothelial Cancer

**DOI:** 10.1155/2017/7830207

**Published:** 2017-07-03

**Authors:** Ryoko Sawada, Yusuke Shinoda, Aya Niimi, Tohru Nakagawa, Masachika Ikegami, Hiroshi Kobayashi, Sakae Tanaka, Yukio Homma, Nobuhiko Haga

**Affiliations:** ^1^Department of Orthopedic Surgery, The University of Tokyo Hospital, Tokyo, Japan; ^2^Department of Rehabilitation Medicine, The University of Tokyo Hospital, Tokyo, Japan; ^3^Department of Urology, Graduate School of Medicine, The University of Tokyo, Tokyo, Japan

## Abstract

Metastasis may occur in any bone but more commonly occurs in the spine, pelvis, or other axial bones. Metastasis in peripheral bones located distal to the elbow or knee, so-called acrometastasis, is rare. Although the mechanism of acrometastasis development is not completely understood, it is thought to be the result of a massive dissemination of cancer cells; thus the prognosis of patients with acrometastasis is relatively poor. Here, we report the case of renal pelvic cancer with multiple acrometastases in both the upper and lower extremities without axial bone metastasis in a 68-year-old man. After two regimens of chemotherapy, he suffered from pain on his wrist and ankle and swelling and hemorrhage of his toe. He had no axial bone metastasis by CT but was diagnosed with multiple acrometastases by plain radiographs. Radiation therapy and disarticulation of the left big toe at the metacarpal-phalangeal joint were performed and his pain and hemorrhage were successfully controlled. Although acrometastasis from renal pelvic cancer is very rare, we should recognize that acrometastasis might occur which exists outside of the CT scanning field.

## 1. Introduction

Metastasis in peripheral bones located distal to the elbow or knee, so-called acrometastasis [1], is rare. Acrometastases are occasionally reported in lung, renal cell, breast, or colon cancer patients; however, there are no reported cases of renal pelvic cancer with acrometastasis. Herein, we report, to the best of our knowledge, the first case of multiple acrometastases arising from renal pelvic cancer.

## 2. Case Report

The patient was a 68-year-old man diagnosed with Stage II left renal pelvic cancer by urine cytology and computed tomography (CT). After undergoing total nephroureterectomy, pathological diagnosis was confirmed as urothelial carcinoma, pT3, G2>G1, ly0, v1, N0, and M0 Stage III. He received 5 cycles of adjuvant chemotherapy (gemcitabine and cisplatin). Lung metastasis appeared after the completion of chemotherapy, whereupon the regimen was changed to docetaxel, ifosfamide, and cisplatin. After 2 cycles of this regimen, the patient underwent partial right lung resection. Pathological diagnosis was metastatic urothelial carcinoma. However, renal dysfunction also progressed; therefore, additional chemotherapy was not performed.

Seventeen months after the right lung resection, new lung and para-aortic lymph node metastases were detected; partial left lung resection was performed, which was pathologically diagnosed as metastasis of urothelial carcinoma, while radiotherapy to the lymph node (50 Gy/25 fractions) was administered. However, multiple lung metastases and subcutaneous metastasis in the abdomen were detected within two months.

**Figure 1:**
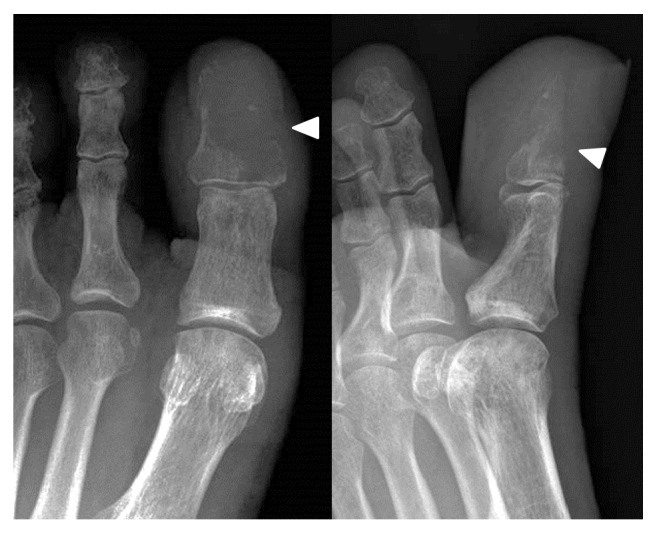
Plain radiographs of the left foot show destruction of the first distal phalanx.

**Figure 2:**
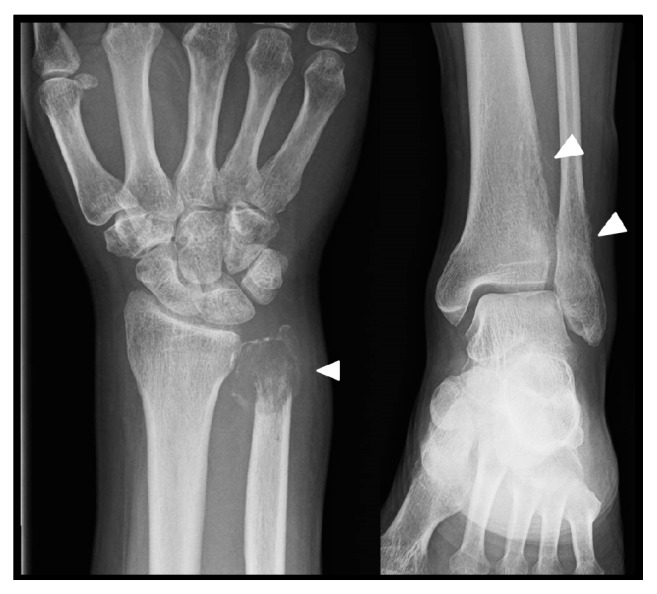
Plain radiographs of right distal ulna and the left lower leg show irregular osteolysis.

**Figure 3:**
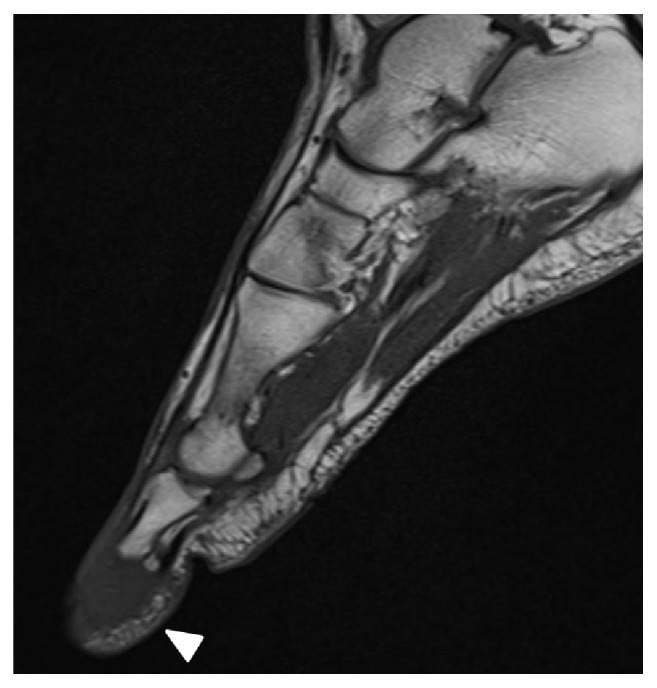
T1-weighted magnetic resonance imaging of the left foot shows low intensity in the first distal phalanx.

Six months after the left lung resection, his left toe became darkly discolored and swollen; he also had pain in his right wrist and left ankle. He visited a nearby orthopedic clinic and was urgently transferred to our hospital because bone metastases were suspected. Plain radiographs revealed osteolytic lesions in the distal end of his right ulna and first distal phalanx of his left foot. Radiographs also showed irregular osteolysis in the cortices of his left distal tibia and fibula, which strongly indicated bone metastases (Figures 1 and 2). On foot magnetic resonance images, bone metastasis of the left big toe was observed spreading across the distal phalanx (Figure 3). Technetium bone scintigraphy also showed abnormal uptake in these areas.

**Figure 4:**
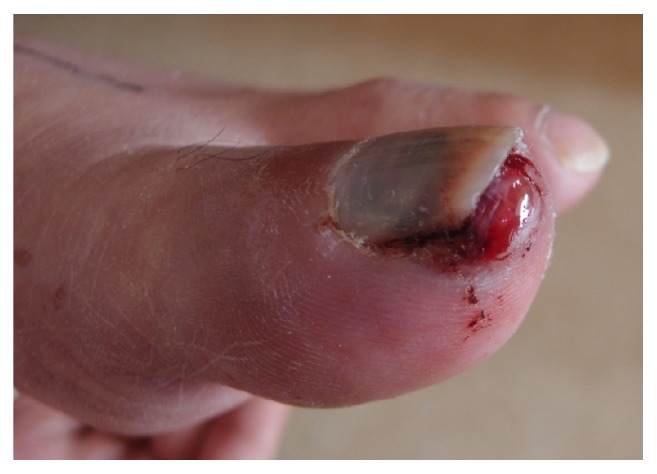
Photograph of the left foot. Subungual hemorrhage from the metastasis, which required a change of dressing on a daily basis, was observed.

Although the patient's toe pain was manageable with painkillers, subungual hemorrhage appeared three days later. As we considered that it would be difficult to control hemorrhaging with radiation therapy alone (Figure 4), we dissected the distal phalanx. Postsurgical pathology showed that the lesion was a metastatic urothelial carcinoma. We also give radiation therapy to his right wrist and his left lower leg (30 Gy/10 fractions). At discharge, the patient could walk with a T-cane and did not have functional pain.

Although the patient was able to spend a good amount of time at home with his family for 2 months, he developed a consciousness disorder owing to multiple brain metastases and died 3 months after the disarticulation surgery.

## 3. Discussion

The common sites of bone metastasis from cancer are axial bones, proximal femur, and proximal humerus. Acrometastasis account for only 0.1% of bone metastases [2–4]. As asymptomatic acrometastases are potentially undetectable because the hands and feet are located outside the periodic CT scanning fields and even most positron emission tomography/CT images, bone metastases distal to the knees and elbows are seldom reported [1, 2, 5]. In this patient, multiple acrometastases were discovered by his symptoms only, even though periodic CT scans were performed. We had not performed PET-CT or bone scintigraphy because European Association of Urology Guidelines recommend cystoscopy and CT scan for following-up upper urinary tract urothelial cell carcinoma [6]. If other bone metastasis had been detected in his axial bone by periodic CT scans, we might have performed PET/CT or bone scintigraphy for surveying other bone metastasis before his symptoms arose.

Lung cancer followed by renal cell, breast, and colon cancers is the most common primary tumors responsible for acrometastasis [4]. Although the mechanism of acrometastasis has not been fully elucidated, it is accepted that bone metastasis, including to the acral regions, occurs hematogenously [2, 4]. It is difficult for disseminated cancer cells to adapt, proliferate, and form metastases in peripheral bones, because of less red marrow content compared to axial bones and lower temperature according to their location closer to surface of the skin [7]. Considering the poor prognosis of patients with acrometastasis [1, 3], massive dissemination of cancer cells may be required [1].

In reported cases of acrometastases, there is little information concerning the existence of other bone metastases in the axial skeleton. However, as massive dissemination of cancer cells may induce acrometastasis, it is natural to assume that such patients would have metastases in the axial bones as well. In our case, it was noteworthy that there were no metastases to the axial bones; moreover, our patient had metastases in the acral regions of both the upper and the lower extremities. Considering that lung cancer is the most frequent malignancy associated with acrometastasis, systemic arterial spread of tumor microemboli may reach the acral skeleton directly if a tumor thrombus accesses the systemic circulation through the left atrium or ventricle [1, 8]. Our patient had multiple lung metastases and had quite a long history of repeated resection. Thus, anterograde systemic tumor embolization might explain the events that transpired in our patient, who had multiple acrometastases in both his hand and foot with no axial bone involvement.

It is reported that the predominant metastatic sites of renal pelvic cancer are the lungs, liver, and bone. Tanaka et al. reported that 5.2% of 402 renal pelvic cancer patients experienced bone metastasis as the primary metastatic site after surgery [9]. However, there are few reported cases of bone metastasis of renal pelvic cancer. Over 90% of primary renal pelvic cancers are urothelial carcinomas, as are the majority of ureteral and bladder cancers. Tsuda et al. reported that only two out of 48 patients with bone metastasis from urothelial carcinoma had acrometastasis [10]. Although there are several case reports of metastases to the foot from bladder cancer [5], this case is likely the first reported case of multiple acrometastases arising from renal pelvic cancer.

Although there is no standard protocol for treatment for acrometastases, treatment is aimed at palliation because of the poor prognosis. Amputation is the most frequent surgical option when the lesion is located in the distal phalanx. Radiation therapy has been attempted also, in order to obtain local control or pain relief [4].

## 4. Conclusion

Acrometastasis might occur from renal pelvic cancer, which cannot be detected by periodical CT scan. We have to listen symptoms of patients carefully, and if acrometastasis is detected, it should be treated rapidly for relieving their pain as their prognosis might be poor.
